# A molecular basis underpinning TRBV28^+^ T-cell receptor recognition of MR1–antigen

**DOI:** 10.1016/j.jbc.2025.110416

**Published:** 2025-06-24

**Authors:** Wael Awad, Nicholas A. Gherardin, Lisa Ciacchi, Andrew N. Keller, Ligong Liu, David P. Fairlie, James McCluskey, Dale I. Godfrey, Jamie Rossjohn

**Affiliations:** 1 Infection and Immunity Program and Department of Biochemistry and Molecular Biology, Biomedicine Discovery Institute, Monash University, Clayton, Victoria, Australia; 2 Department of Microbiology and Immunology, The Peter Doherty Institute for Infection and Immunity at the University of Melbourne, Melbourne, Victoria, Australia; 3 Institute for Molecular Bioscience, University of Queensland, Brisbane Queensland, Australia; 4 Institute of Infection and Immunity, Cardiff University, School of Medicine, Heath Park, Cardiff, UK

**Keywords:** antigen presentation, MAIT, MHC-related molecule 1, protein structure, TCR

## Abstract

Mucosal-associated invariant T (MAIT) cells express a TRAV1-2^+^ T-cell receptor (TCR) that recognizes microbial vitamin B2 derivatives presented by the major histocompatibility complex class I–related molecule (MR1). Most MAIT TCRs incorporate a biased TCR-**β** repertoire, predominantly TRBV20-1 and TRBV6, but some utilize other *trbv* genes, including TRBV28. A second conserved, albeit less frequent TRAV36^+^ TRBV28^+^ T-cell population exhibits MAIT-like phenotypic features but use a markedly distinct mode of MR1–antigen (Ag) recognition compared with MAIT TCR–MR1 binding. Nevertheless, our understanding of how differing TCR gene usage results in altered MR1 binding modes remains incomplete. Here, binding studies demonstrated differential affinities and Ag specificities between TRBV6^+^ and TRBV28^+^ MR1-restricted TCRs. Alanine-scanning mutagenesis on the TRAV36–TRBV28 TCR revealed a strong dependence on germline-encoded residues within the highly selected complementarity-determining region 3**α** loop, similar to TRAV1-2–TRBV6 TCRs, and further alanine-scanning mutagenesis experiments demonstrate differential energetic footprints by these TCRs atop MR1. We determined the crystal structure of a MAIT TRAV1-2–TRBV28^+^ TCR–MR1–5-OP-RU ternary complex. This structure revealed a docking mode conserved amongst other TRAV1-2^+^ MAIT TCRs, with the *trbv28*-encoded TCR-**β** chain adopting highly distinct docking modes between the TRAV1-2^+^ and TRAV36^+^ TCRs. This indicates that the TCR-α chain dictates the positioning and role of the TCR-**β** chain. Taken together, these findings provide new molecular insights into MR1–Ag-driven selection of paired TCR-**α** and TCR-**β** chains.

The major histocompatibility complex (MHC)–related protein 1 (MR1) is an evolutionarily conserved MHC class I–like molecule that presents small metabolite antigens (Ags) to αβ and γδ T cells, including mucosal-associated invariant T (MAIT) cells (1, 2, 3). MAIT cells are highly abundant in humans, representing up to 10% of T cells in blood (4), and are further enriched in tissues such as the liver (5). The predominant role of MAIT cells is thought to be antimicrobial immunity, though emerging studies suggest that they likely also play roles in diverse inflammatory and autoimmune diseases as well as cancer (6, 7). MAIT cell antimicrobial immunity is largely mediated by recognition of conserved microbial riboflavin metabolites that are captured and presented by MR1 at the surface of infected cells, flagging a molecular signature of infection (1). Here, a biosynthetic intermediate of microbial riboflavin (vitamin B2) synthesis known as 5-amino-6-(1-D-ribitylamino)uracil (5-A-RU), produced by a diverse range of bacteria and yeast, reacts nonenzymatically with ubiquitous small chemical scaffolds, resulting in the production of unstable ribityl-pyrimidines such as 5-(2-oxopropylideneamino)-6-D-ribitylaminouracil (5-OP-RU) as well as the ribityl-lumazines such as 7-hydoxy-6-methyl-8-D-ribityllumazine (RL-6-Me-7-OH) (8), both of which are captured within the A′ pocket of MR1 (9). This MR1–Ag complex is in turn detected by the evolutionarily conserved MAIT T-cell receptor (TCR) (10).

In humans, the MAIT TCR typically comprises a TCR-α chain encoded by the *trav1-2* gene recombined with either *traj33*, *traj12*, or *traj20*, with little diversity in the complementarity-determining region 3 (CDR3α) loop (3, 11). This TCR-α chain pairs with TCR-β chains utilizing a repertoire highly enriched for the *trbv6-1*, *trbv6-4*, and *trbv20* genes paired to diverse *trbj* genes, with a hypervariable CDR3β loop (12). The salient features of MR1–Ag recognition by the MAIT TCR are reasonably well understood (9). Here, the TCR docks MR1 in an orthogonal docking mode, straddling the α1 and α2 helices of MR1. This positions the invariant CDR3α loop at the opening of the A′ pocket of MR1, allowing the formation of an interaction triad where the Tyr95α residue within the CDR3α loop forms a hydrogen bond with the 2′-OH group of the ribityl tail of 5-A-RU-based microbial Ags, as well as MR1-Tyr152 (8, 10, 13, 14, 15). Indeed, any modifications to the ligand that disrupts the interaction triad reduces or abolishes MAIT cell activation (16). Moreover, variation in the hypervariable CDR3β loop, which sits adjacent to the invariant CDR3α loop within proximity of the Ag-binding A′ pocket, can fine-tune MAIT TCR recognition of riboflavin-based and other MR1-presented metabolites (17, 18, 19). Beyond the microbial-derived pyrimidine and lumazine ribityl Ags, the Ag-binding pocket of MR1 can accommodate chemically diverse ligands, including natural, environmental, drugs, and drug-like molecules (18, 20, 21, 22) as well as self-derived ligands (23, 24, 25, 26). Examples include the pterin-based vitamin B9 (folate)–derived metabolites, 6-formyl pterin (6-FP) (1), a photodegradation product of folate, and its synthetic analog acetyl-6-FP (Ac-6-FP) (14). Many MR1 ligands are characterized by their ability to be captured and stabilized by MR1 *via* formation of a covalent imine (Schiff base) with Lys43 within the A′ pocket, and the reactive aldehyde or ketone moieties of the ligand (1, 9, 27), though not all ligands form this Schiff base, including the recently described bile-acid metabolites (24, 28).

Previous studies using MR1–Ag tetramers have revealed diverse populations of TRAV1-2^-^ MR1-restricted αβ T cells in humans, expressing a broad range of *trav* and *trbv* genes (17, 29, 30, 31). This includes a second albeit infrequent population of T cells incorporating TRAV36–TRBV28-biased TCR usage. This population of TRAV36–TRBV28^+^ MR1-restricted T cells maintains the core features of MAIT cells, including restriction to MR1-presenting ribityl Ags, a MAIT-like cell surface phenotype with high levels of CD161, IL-18Rα, and CD26, CD8, or CD4^-^CD8^-^ double-negative coreceptor expression, and an MAIT-like transcription factor profile of PLZF^+^, RORγt^+^, and T-bet^INT^ (17, 29). Like the classical TRAV1-2^+^ MAIT TCR, TRAV36^+^ TCRs express a highly restricted TCR-α chain, recombining *trav36* with *traj34* or *traj37*, resulting in a germline-encoded CDR3α loop of invariant sequence and length. Unlike TRAV1-2^+^ MAIT TCRs however, TRAV36^+^ MAIT TCRs furnish a highly restricted TCR-β chain, pairing *trbv28* with *trbj2–5* to generate CDR3β loops with similar sequence diversity and length (29). Structural analysis of a TRAV36^+^ MAIT TCR, clone “MAV36,” revealed a highly distinct mechanism of MR1–Ag recognition, whereby Asn29α of the CDR1α loop made direct contact with the ribityl tail of 5-OP-RU (17). *Trbv28*-encoded residues of the TCR-β chain made numerous contributions to MR1 docking (17); however, why there is less diversity in the TCR-β chain repertoire of TRAV36^+^ MAIT TCRs compared with TRAV1-2^+^ MAIT TCRs remains unclear.

Here, we compared TRAV1-2^+^ and TRAV36^+^ MAIT TCR recognition of MR1–Ag. Alanine-scanning mutagenesis of the MAV36 TCR was performed to understand the importance of the CDR3α and CDR3β loops in mediating Ag recognition. Binding studies were subsequently undertaken to compare the kinetics of MR1–Ag complex binding between distinct MR1-restricted TCRs. Furthermore, the crystal structure of a TRAV1-2^+^ TRBV28^+^ TCR, clone “MBV28,” in complex with MR1–5-OP-RU, was determined, allowing direct comparison to the TRAV36^+^ TRBV28^+^ TCR and TRAV1-2^+^ TRBV6-1 TCR–MR1–Ag complexes. Collectively, our findings enhance our understanding of how diverse TCR usage manifests in altered patterns of MR1–Ag recognition.

## Results

### MR1-reactive TCRs display differential affinities for MR1-Ags

**Figure 1:**
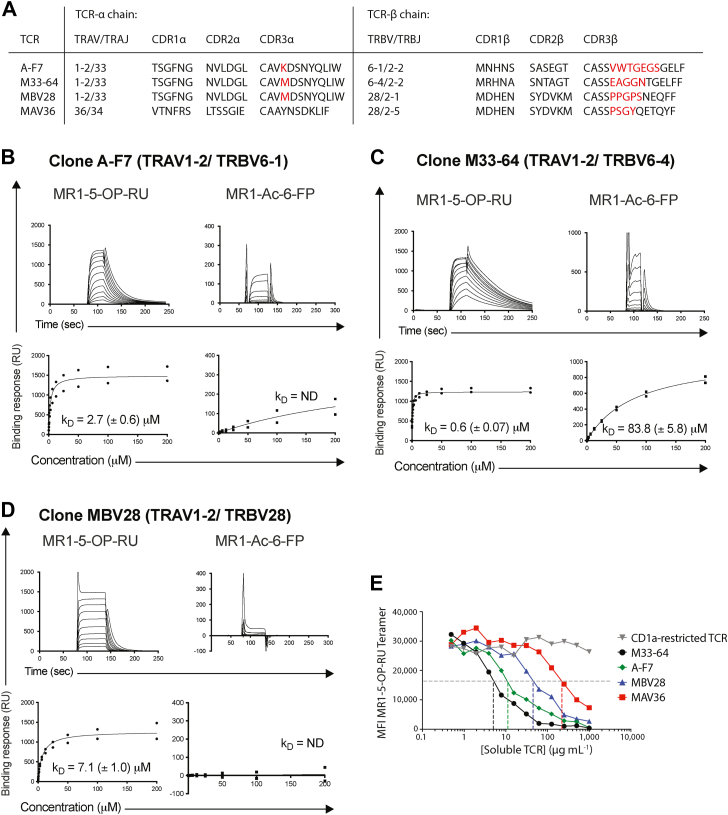
**MR1 reactivity by diverse T-cell receptors (TCRs).***A*, the table showing TCR sequences of clones used in this study. *Red residues* are nongermline encoded. *B*–*D*, steady state binding affinities of soluble TCRs for MR1–antigen complexes. The displayed SPR sensorgrams are of a single series of concentrations of the (*B*) A-F7, (*C*) M33-64, and (*D*) MBV28 TCRs, against MR1–5-OP-RU (*left panel*) and MR1–Ac-6-FP (*right panel*). SPR experiments were conducted duplicate in two independent experiments (n = 2). Steady-state *K*_*D*_ values represent mean (of the duplicate runs) ± SEM from two independent experiments. SPR sensograms (*top*) and equilibrium curves (*bottom*) were prepared in GraphPad Prism. For equilibrium curves, data points from both independent experiments are presented with no error bars. Averaged steady-state (*K*_*D*_) values are shown for each TCR *versus* MR1–5-OP-RU, Ac-6-FP, respectively. *E*, line graph showing a tetramer inhibition assay, which measures the relative ability of graded concentrations of soluble TCR (CD1a-restricted TCR [clone BK6] control, typical MAIT TCRs: A-F7, M33–64, and MBV28 TCR, and atypical MR1-restricted TCR: MAV36) to inhibit MR1–5-OP-RU tetramer staining of the Jurkat.M33-64 cell line. Each point represents the median of duplicate wells. The graph is representative of two independent experiments. *Dotted lines* denote the half maximum tetramer median fluorescence intensity (MFI). Ac-6-FP, acetyl-6-formyl pterin; MAIT, mucosal-associated invariant T; MR1, major histocompatibility complex class I–related molecule; ND, not determined; 5-OP-RU, 5-(2-oxopropylideneamino)-6-D-ribitylaminouracil; RU, response unit; SPR, surface plasmon resonance.

To establish how various MR1-restricted TCRs bind to MR1-Ags, a panel of soluble TRAV1-2^+^ and a TRAV36^+^ MR1–restricted TCR was generated. This included TCRs from clones A-F7 (TRAV1-2–TRBV6-1), M33-64 (TRAV1-2–TRBV6-4), MBV28 (TRAV1-2–TRBV28), and MAV36 (TRAV36–TRBV28) (32) (Fig. 1*A*). Surface plasmon resonance (SPR) experiments were performed to examine the specificities and determine the steady state binding affinities (*K*_*D*_) of A-F7, M33-64, and MBV28 TCRs against MR1–5-OP-RU or MR1–Ac-6-FP ligands (Fig. 1, *B*–*D*). The MR1 autoreactive M33-64 TCR binds to MR1–5-OP-RU and MR1–Ac-6-FP with *K*_*D*_ 0.6 and 82 μM, respectively, whereas A-F7 TCR binds only to MR1–5-OP-RU (*K*_*D*_ ∼3 μM) (Fig. 1, *B* and *C*) as previously published (32). The MBV28 TCR exhibited twofold lower affinity of *K*_*D*_ 7.1 μM to MR1–5-OP-RU compared with A-F7 TCR and did not bind to MR1–Ac-6-FP (Fig. 1*D*).

Next, to complement SPR experiments, tetramer inhibition assays were performed to measure the relative ability of graded concentrations of soluble TCRs to inhibit MR1–5-OP-RU tetramer staining of Jurkat T-cell lines expressing the MAIT TCR clone M33-64 (Jurkat.M33–64) (Fig. 1*E*). As anticipated, the negative control, CD1a-restricted TCR clone BK6, did not inhibit MR1–5-OP-RU tetramer staining at any dose tested. The series of MAIT TCRs inhibited staining in a dose-dependent manner with approximate 50% inhibition achieved in a hierarchy among the TCR clones: M33-64 > A-F7 > MBV28 > MAV36 (Fig. 1*E*), consistent with SPR-based affinity measurements in this study and as formerly published (17).

### Energetic basis underpinning TCR Ag recognition

**Figure 2:**
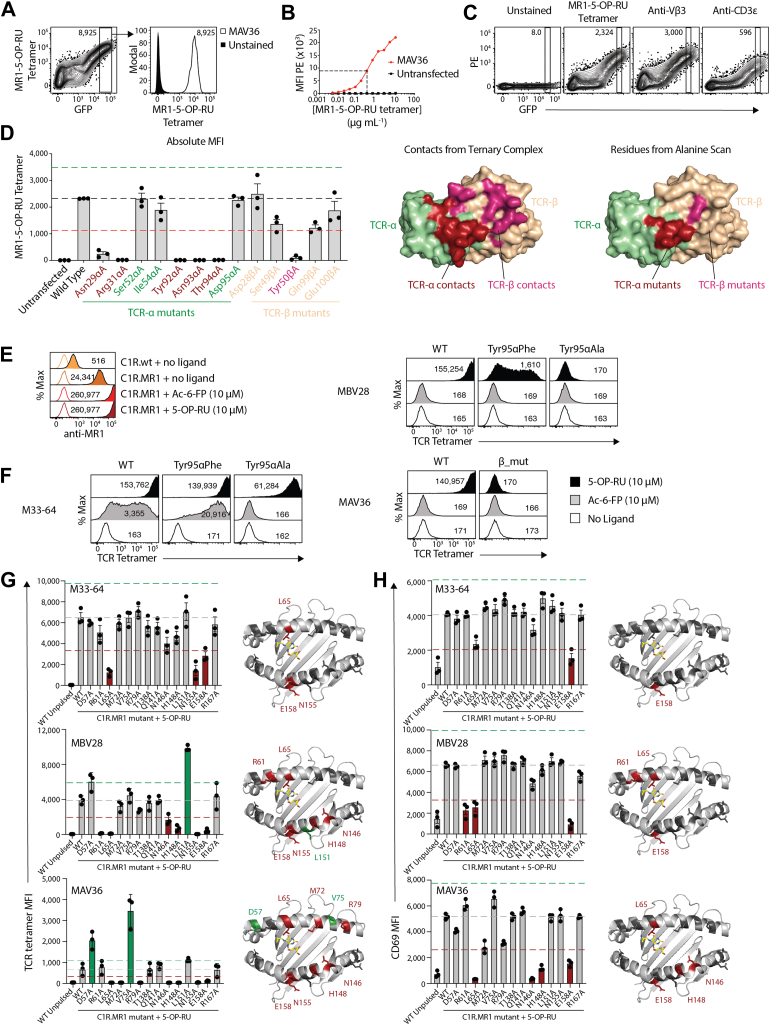
**Alanine-scanning mutagenesis.***A*, flow cytometric contour plots (*left*) showing MR1–5-OP-RU tetramer staining on HEK293T cells transiently transfected with CD3 subunits and the wildtype MAV36 TCR. The median fluorescent intensity (MFI) is at the top of the plot. The gated GFP^+^ population is shown in the histogram on the *right*, overlaying the same gate from stained and unstained samples. *B*, line graph showing MR1–5-OP-RU tetramer staining intensity from gating depicted in (*A*) on MAV36 TCR-expressing HEK293T (*red*) or untransfected (*black*), at titrating doses of MR1–5-OP-RU tetramers. The *dotted line* shows the selected dose. *C*, flow cytometric contour plots showing staining profiles of phycoerythrin (PE)-labeled MR1–5-OP-RU tetramers, anti-Vβ3 antibodies, or anti-CD3ε antibodies. Gates are set in a consistent window of GFP expression, and the numbers at the *top* refer to the MFI of the gated population. *A*–*C*, representing n = 2 individual experiments each. *D*, *left*, bar graphs showing the absolute MFI of HEK293T cells transfected to express with CD3 subunits and MAV36 alanine mutants and stained with MR1–5-OP-RU tetramers at the selected concentration derived from (*B*). *Right*, surface representations of the MAV36 TCR (Protein Data Bank entry: 5D7K) comparing residues involved in contacts with MR1–5-OP-RU identified from the ternary complex crystal structure, and residues that knockdown staining in the alanine scan mutagenesis study here. TCR-α chain residues are denoted in *green*, and TCR-β chain residues denoted in *wheat*, with mutations affecting binding shown in *red* and *pink* for the TCR-α and TCR-β, respectively. *E* and *F*, flow cytometric histogram overlays showing (*E*) anti-MR1 clone 26.5 antibody (*solid*) overlayed on isotype control (clear), or (*F*) TCR tetramer staining on C1R.MR1 cells pulsed for 5 h with 5-OP-RU or Ac-6-FP. Data in (*E*) and (*F*) are representative of two independent experiments. *G*, bar graphs (*left*) showing TCR tetramer staining MFI on C1R cell lines pulsed for 3 h with 5-OP-RU. Cartoon images (*right*) of the MR1 antigen–binding cleft highlighting the location of amino acid residues corresponding to mutant cell lines, which induced a 50% increase (*green*) or decrease (*red*) in tetramer staining relative to WT. *H*, bar graphs (*left*) showing CD69 expression on SKW-3.TCR cell lines after 20 h coculture with C1R cells incubated with 5-OP-RU. *Cartoon images* (*right*) of the MR1–antigen–binding cleft highlighting the location of amino acid residues corresponding to mutant cell lines, which induced a 50% increase (*green*) or decrease (*red*) in CD69 upregulation relative to WT. Individual datapoints in (*D*), (*G*), and (*H*) represent mean of duplicate wells from n = 3 independent experiments with error bars depicting SEM. Ac-6-FP, acetyl-6-formyl pterin; HEK293T, human embryonic kidney 293T cell line; MR1, major histocompatibility complex class I–related molecule; 5-OP-RU, 5-(2-oxopropylideneamino)-6-D-ribitylaminouracil; TCR, T-cell receptor.

To probe the molecular basis for differential MR1–Ag reactivity by TRAV1-2^+^ and TRAV36^+^ TCRs, we next undertook a series of alanine-scanning mutagenesis–based assays. TRAV36^+^ MAIT TCRs express a semi-invariant TCR-α chain and restricted TCR-β chain, namely *trbv28* with *trbj2–5* with only limited nongermline encoded-nucleotide additions at the CDR3β V–J junction (29). While we established that the TRAV36–TRBV28 TCR adopted a differing docking topology atop MR1 when compared with TRAV1-2 MAIT TCR–MR1 recognition (17), it remained unclear which residues of the MAV36 TCR were key in mediating binding to MR1. We therefore performed alanine-scanning mutagenesis followed by flow cytometry–based binding studies on five residues on the TCR-α chain (Asn29α, Arg31α, Tyr92α, Asn93α, and Thr94α) and four residues on the TCR-β chain (Ser49β, Tyr50β, Gln99β, and Glu100β) that were at the TCR–MR1 binding interface, as well as control residues that were distal to the TCR–MR1 interface (Ser52α, Ile54α, Asp95α, and Asp28β) (17). The selected mutants had particular focus on not only *traj*/*trbj* gene–encoded residues but also included key TRAV–TRBV residues that either interacted with the Ag (Asn29α) or appeared to modulate TRBV28 binding (Ser49β and Tyr50β). Plasmids encoding the MAV36 TCR and subunits of the human CD3 complex were transiently transfected into human embryonic kidney 293T (HEK293T) cells, resulting in surface expression of MAV36 TCR, correlating with GFP expression and permitting MR1–5-OP-RU tetramer staining (Fig. 2*A*). Titration of MR1–5-OP-RU tetramers on these transfected cells found that 0.5 μg/ml provided a nonsaturating dose of tetramer that would be more sensitive to altered binding strength relative to a saturating dose, while still providing robust tetramer staining intensity (Fig. 2*B*). Beyond tetramer staining, TCR levels could also be measured by separate stains with anti-Vβ3 antibodies, which bind the *trbv28*-encoded β-chain, as well as anti-CD3ε, thus when comparing MR1–5-OP-RU tetramer staining by mutant TCRs transfected separately, the GFP gate can be normalized for TCR expression using either of these markers (Fig. 2*C*). Accordingly, similar transient transfections were performed with single alanine mutant MAV36 TCRs and subsequently stained with MR1–5-OP-RU tetramers, anti-Vβ3, or anti-CD3ε antibodies (Fig. 2*D*). For each mutant, GFP gates were set based on a defined median fluorescence intensity (MFI) of phycoerythrin (PE) using the Vβ3-stained cells, and this gate then applied to the MR1–5-OP-RU tetramer–stained cells, thus normalizing the levels of surface expression between mutants. Mutation of Asn29α and Arg31α in the CDR1α loop ablated MR1–5-OP-RU tetramer staining, whereas Ser52α and Ile54α had little to no impact. This aligns with published crystallographic data (17), in which Asn29α directly contacts the ribityl moiety of 5-OP-RU, whereas Arg31α forms a salt bridge with Glu149 of MR1. On the other hand, Ser52α and Ile54α are solvent-exposed residues distal to the MR1–Ag complex and are not involved in TCR-binding interactions. In this crystal structure, the CDR3α loop made contacts with both the CDR1α and CDR3β loops, as well as MR1, and may act to stabilize CDR1α to permit direct Ag recognition. Moreover, these key contacts are mediated by the conserved germline-encoded amino acids of TRAJ34 and TRAJ37. In line with this, three of four single point mutations in the *traj*-encoded residues of the CDR3α loop ablated MR1–5-OP-RU tetramer staining, including Tyr92α, Asn93α, and Thr94α, whereas Asp95α, which sits further from the Ag-binding pocket, had no impact on MR1–5-OP-RU tetramer staining. Upon mutation of TCR-β chain residues, as expected, mutation of Asp28β, which sits distal to the MR1–Ag complex, had no effect on MR1–5-OP-RU tetramer staining. Mutation of Ser49β reduced MR1–5-OP-RU tetramer staining by approximately 50%, whereas mutation of Tyr50β, which makes a series of interactions with the α1 helix of MR1, completely ablated staining. To understand why the *trbj2–5* gene is so highly conserved, the CDR3β mutations included the two *trbj*-encoded residues in close proximity to the Ag-binding cleft, Gln99β and Glu100β; however, Gln99β only reduced MR1–5-OP-RU tetramer staining by approximately 50%, and Glu100β had only a moderate effect at best. Thus, there appears to be a greater requirement for the *traj*-encoded residues of CDR3α relative to the *trbj*-encoded residues of CDR3β to enable MR1 binding. Next, we generated TCR tetramers for M33-64, MBV28, and MAV36 TCRs, as well as mutants thereof (Fig. S1). When MR1-overexpressing cells (C1R.MR1) were treated with 5-OP-RU or Ac-6-FP, cell surface MR1 was increased, as demonstrated by increased staining with an anti-MR1 antibody (Fig. 2*E*). In response to 5-OP-RU, all three wildtype TCRs bound strongly, whereas none bound to untreated cells (Fig. 2*F*). When treated with Ac-6-FP, both MBV28 and MAV36 failed to bind, highlighting their Ag specificity, whereas M33-64 bound weakly, in line with the MR1 autoreactivity inherent to this TCR. Both TRAV1-2+ MAIT TCRs were also mutated at Tyr95α to either phenylalanine or alanine. Notably, while MBV28 Tyr95αAla failed to bind in any condition, the M33-64 Tyr95αAla maintained its 5-OP-RU reactivity, albeit with reduced intensity. Mutation to Tyr95αPhe instilled weak 5-OP-RU reactivity for MBV28, whereas this mutation maintained strong reactivity to 5-OP-RU and moderately enhanced Ac-6-FP reactivity for M33-64, further underscoring the MR1 autoreactivity of M33-64. To probe the dependence on CDR3β for TRAV36^+^ MAIT TCRs, we generated a mutant TCR in which the CDR3β from the MAV36 TCR was swapped with CDR3β of the MBV28 TCR, noting that both these TCRs otherwise used TRBV28. This mutant TCR failed to bind 5-OP-RU-treated C1R.MR1 cells, demonstrating that a permissive hypervariable CDR3β is required for TRAV36 TCRs. These tetramers were then used to stain a panel of C1R.MR1 point mutant cell lines in which amino acids spanning the α1 and α2 helices of MR1 had been mutated to alanine (Fig. 2*G*) (33). While M33-64 had reduced binding in response to Leu65Ala, Asn155Ala, and Glu158Ala mutations, MBV28 had an additional dependency on Arg61, Asn146, and His148, further emphasizing the MR1 autoreactivity of the M33-64 TCR. MAV36 had a distinct pattern of reactivity spanning a wider, more central portion of MR1, with knockdown in tetramer binding in response to Leu65Ala, Met72Ala, Arg79Ala, Asn146Ala, His148Ala, Asn155Ala, and Glu158Ala mutations. Moreover, Asp57Ala and Val75Ala resulted in enhanced TCR tetramer binding (Fig. 2*G*). We also undertook complementary activation–based assays, coculturing SKW-3 cell lines expressing each of the TCRs with the C1R.MR1 mutants and 5-OP-RU, measuring CD69 on the SKW-3 cells as a readout of TCR signaling and activation (Fig. 2*H*). Activation of all three TCR^+^ cell lines by mutants showed a broadly similar footprint to the tetramer staining experiments, albeit with reduced or no sensitivity to some MR1 mutations, which probably reflects enhanced avidity in the context of cellular interactions in comparison to TCR tetramer staining. Thus, M33-64 still showed sensitivity to Glu158Ala but was no longer affected by Asn155Ala mutation and was less affected by Leu65Ala mutation. MBV28 was dependent on Arg61, Leu65, and Glu158 but was no longer affected by Asn146Ala, His148Ala, Leu151Ala, and Asn155Ala mutations. MAV36 was inhibited by Leu65Ala, Asn146Ala, His148Ala, and Glu158Ala but less dependent on Asn155 and a number of residues on the α1 helix, namely Asp57, Met72, Val75, and Arg79. Taken together, these collective results highlight differential energetic footprints on MR1 for TRAV1-2 and TRAV36 TCRs.

### Overview of the TRAV1-2–TRBV28^+^ TCR–MR1–5-OP-RU ternary complex

**Figure 3:**
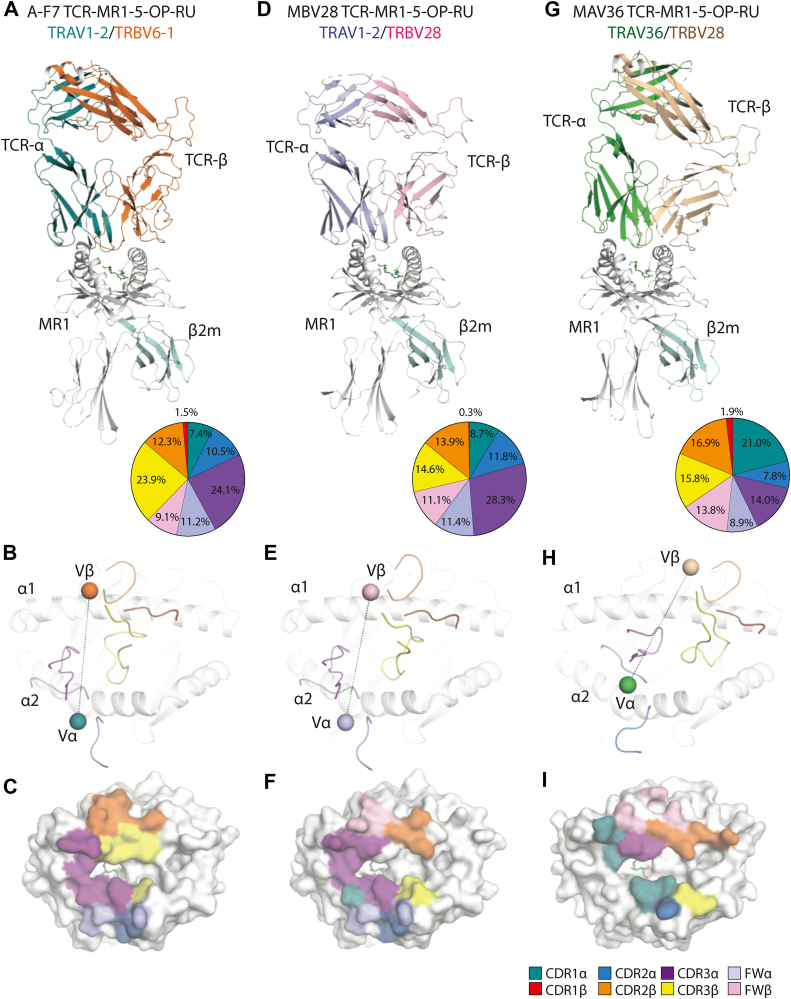
**Structural comparison of ternary complexes of TRBV6-1^+^ and TRBV28^+^ TCRs with MR1–5-OP-RU.** Crystal structures of ternary complexes. *A–C*, A-F7 (TRAV1-2/TRBV6-1) TCR–MR1–5-OP-RU (PDB entry: 6PUC). *D*–*F*, MBV28 (TRAV1-2/TRBV28) TCR–MR1–5-OP-RU. *G*–*I*, MAV36 (TRAV36/TRBV28) TCR–MR1–5-OP-RU (PDB entry: 5D7L). *A*, *D*, and *G*, *top panels* are cartoon representations of the ternary complexes. A-F7 TCRα, *dark teal*; MBV28 TCRα, *light blue*; MAV36 TCRα, *green*; A-F7 TCRβ, *orange*; MBV28 TCRβ, *light pink*; and MAV36 TCRβ, *wheat*. The MR1 heavy chain and β2-microglobulin (β2m) molecules are colored *white* and *pale cyan*, respectively, and 5-OP-RU is presented as *green sticks*. *Pie charts* represent the relative contribution of each segment of the TCRs, A-F7 (*A*), MBV28 (*D*), and MAV36 (*G*) to the buried surface area (BSA) directed against the MR1–5-OP-RU complex. The corresponding complementarity determining region (CDR) loops, namely CDR1α, CDR2α, CDR3α, CDR1β, CDR2β, and CDR3β, are shown in *teal*, *sky-blue*, *purple*, *red*, *orange*, and *yellow*, respectively, and the framework (FW) residues of the α- and β-chains are shown in *light blue* and *light pink*, respectively. The *middle panels* show the CDR loops of the A-F7 (*B*), MBV28 (*E*), and MAV36 (*H*) TCRs docking onto MR1. Each docking angle is shown as a *black dashed line* connecting the center of mass (COM) of Vα with the COM of Vβ, which are represented as a *sphere* colored consistent with chain colors in the *upper panels*. The *lower panels* illustrate the respective A-F7 (*C*), MBV28 (*F*), and MAV36 (*I*) TCR footprints on the molecular surface of MR1–5-OP-RU. The atomic footprint is colored based on the TCR segment making contact. A-F7, Ac-6-FP, acetyl-6-formyl pterin; MR1, major histocompatibility complex class I–related molecule; 5-OP-RU, 5-(2-oxopropylideneamino)-6-D-ribitylaminouracil; PDB, Protein Data Bank; TCR, T-cell receptor.

To gain insight into the molecular basis for binding of TRBV28^+^ TCRs to MR1–5-OP-RU in the context of pairing with TRAV1-2 or TRAV36 chains, the crystal structure of the MBV28 (TRAV1-2–TRBV28) TCR–MR1–5-OP-RU ternary complex was determined to 3.1 Å resolution, then compared with the published MAV36 (TRAV36–TRBV28) TCR–MR1–5-OP-RU and A-F7 (TRAV1-2–TRBV6.1) TCR–MR1–5-OP-RU structures (Fig. 3 and Table 1). Consistent with all published MAIT TCR–MR1–5-OP-RU complexes (14), the 5-OP-RU ligand in the MBV28 TCR–MR1–5-OP-RU complex was sequestered within the A′ pocket and formed a Schiff-base covalent bond with MR1-Lys43.

**Table 1:** Data processing and refinement statistics

Parameters	MBV28 TCR–MR1–5-OP-RU
Wavelength (Å)	0.9537
Resolution range	46.83–3.10 (3.20–3.10)
Space group	P 41 21 2
Unit cell	
a, b, c (Å)	111.35, 111.35, 206.46
α, β, γ (°)	90, 90, 90
Total reflections	207,643 (34,632)
Unique reflections	24,316 (2385)
Multiplicity	3.00
Completeness (%)	99.91 (99.96)
CC(1/2)	0.977 (0.625)
Mean I/sigma(I)	2.19
Wilson *B*-factor	64.36
*R*-merge	0.220
*R*pim	0.143
*R*-work	0.2233 (0.2942)
*R*-free	0.2532 (0.3193)
Number of nonhydrogen atoms	5889
Macromolecules	5745
Ligands	40
Solvent	104
Protein residues	735
RMSD (bonds)	0.002
RMSD (angles)	0.50
Ramachandran favored (%)	98.03
Ramachandran allowed (%)	1.83
Ramachandran outliers (%)	0.14
Average *B*-factor	78.54
Macromolecules	79.14
Ligands	61.07
Solvent	52.55

Statistics for the highest-resolution shell are shown in parentheses. Rmerge = ∑h∑Iih − <Ih>/∑h∑I<Ih>, where <Ih> is the mean intensity of the observations Iih of reflection h. R-factor = ∑(Fobs − Fcalc)/∑Fobs; Rfree is the R-factor for a subset (5%) of reflections that was selected prior to refinement calculations and not included in the refinement.

**Figure 4:**
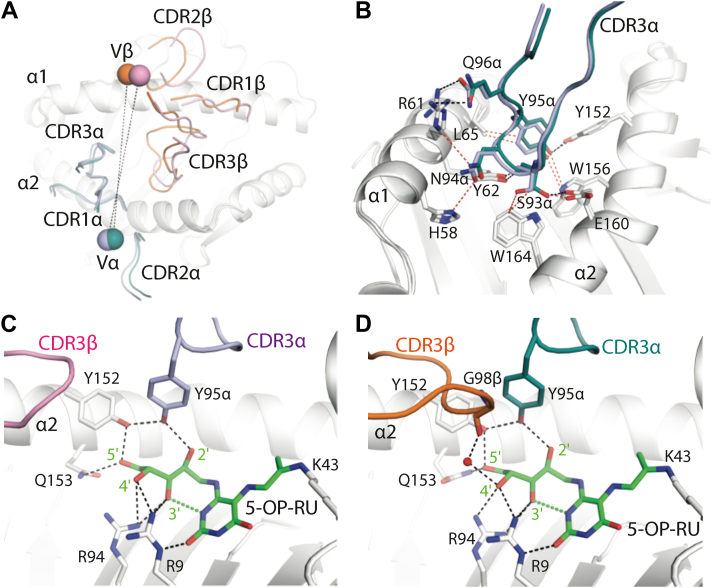
**Interface comparison of TRBV6-1^+^ and TRBV28^+^ MAIT TCRs in complex with MR1–5-OP-RU.***A*, superposition of CDR loops from the A-F7 TCR (CDRα, *dark teal*; CDRβ, *orange*) and the MBV28 TCR (CDRα, *light blue*; CDRβ, *light pink*), and superposition of the center of masses of TCR variable α- and β-chain domains above MR1 (*white cartoon*) for A-F7 (Vα, *dark teal blue sphere*; Vβ, *orange sphere*) and MBV28 (Vα, *light blue sphere*; Vβ, *light pink sphere*) TCRs, as connected by a *dashed line.B*, Superposition of CDR3α loops of the A-F7 and MBV28 (CDR loops as colored in *A*) TCRs interacting with MR1. *C*, interactions of the CDR3α of MBV28 TCR with 5-OP-RU ligand and MR1, including the location of the CDR3β. *D*, interactions of the CDR3α of the A-F7 TCR with 5-OP-RU and MR1 as well as the water-mediated contact between CDR3β and 5-OP-RU. Hydrogen bonds are represented as *black dashes*; salt bridges as *yellow dashes*; and van der Waals contacts are represented as *dotted red lines*. CDR, complementarity-determining region; MR1, major histocompatibility complex class I–related molecule; 5-OP-RU, 5-(2-oxopropylideneamino)-6-D-ribitylaminouracil; TCR, T-cell receptor.

The MBV28 TCR docked ∼82° to the main axis of the MR1–Ag binding cleft, with the TCR α- and β-chains positioned atop the α2- and α1-helices of MR1, respectively (Fig. 3). The buried surface area (BSA) at the interface between MBV28 TCR and MR1–5-OP-RU was ∼1030 Å^2^, which aligns well with values for other TRAV1-2^+^ MAIT–MR1–5-OP-RU ternary complexes (1050–1200 Å^2^) (Fig. 3) (34). Although the α- and β-chains of A-F7 and MAV36 TCR contribute almost equally to the BSA at the TCR–MR1–Ag interface; the α- and β-chains of the MBV28 TCR contributed ∼60% and ∼40%, respectively, which is attributed to the altered docking modality of the MBV28 TCR β-chain to MR1. Specifically, the center of gravity of the MBV28 TCR β-chain was displaced by ∼2.2 Å toward the F′ pocket compared with the corresponding β-chain of the TRAV1-2–TRBV6-1^+^ A-F7 TCR (Fig. 4*A*). Yet, the MBV28 TCR adopted a docking mode largely resembling that of other TRAV1-2^+^ MAIT TCRs, and distinct to that of TRAV36^+^ TRBV28^+^ TCRs, despite the shared use of TRBV28 (Fig. 3, *A*, *D* and *G*).

### TRAV1-2–TRBV28^+^ TCR recognition of microbial Ags depends on convergent TRAV1-2 TCR-**α** chain interactions

The TRAV1-2–TRAJ33^+^ of the MBV28 TCR α-chain docks similarly in both MBV28 and A-F7 TCRs, contributing to 60% of the MBV28 TCR BSA to the interface (∼615 Å^2^), where the CDR3α contributed the most to this interaction at 28.3%, followed by CDR2α (11.8%), α-framework (FWα) region (11.4%), and last, CDR1α with 8.7% BSA (Fig. 3*D*). The MBV28 TCR comprises a unique set of *trav1-2*-encoded CDR1α residues, Gly28α, Phe29α, and Asn30α as well as CDR2α residues, Val50α and Leu51α, which mediate conserved contacts with MR1 (Table 2). Further, the *traj33*-encoded CDR3α motif residues, ^93^Ser-Asn-Tyr-Gln^96^, of the MBV28 TCR forms conserved interactions with MR1 residues, including His58, Arg61, Tyr62, Leu65, Tyr152, Trp156, and Glu160 (Fig. 4*B*). Akin to the A-F7 TCR–MR1–5-OP-RU complex, the conserved Tyr95α of the MBV28 TCR formed a hydrogen bond with 2′OH group of 5-OP-RU and Tyr152 of MR1 α1-helix, thus forming the “*interaction triad*” (Fig. 4, *C* and *D*). The positioning of the ribityl tail of 5-OP-RU was governed by polar interactions mediated by MR1 residues, Arg9, Arg94, Tyr152, and Gln153 (Fig. 4*C*). Collectively, the MBV28 TCR α-chain TRAV1-2 interactions with MR1 were conserved when compared with the published MAIT TRAV1-2^+^ TCR footprints on MR1 (14, 17, 34).

**Table 2:** Contacts of MBV28 TCR with MR1–5-OP-RU

TCR gene	TCR residue	MR1	Bond type
CDR1α	Gly28	Glu160	VDW
	Phe29	Asn155 & Glu160	HB
	Phe29	Glu160	VDW
	Asn30	Tyr152, Trp156, & Glu160	VDW
CDR2α	Val50	Leu151, Tyr152, Asn155	VDW
	Leu51	Leu151, Lys154, & Asn155	VDW
CDR3α	Ser93	Tyr62	HB
	Ser93	Tyr62, Glu160, & Trp164	VDW
	Asn94	His58, Arg61, & Tyr62	VDW
	Tyr9	Tyr152	HB
	Tyr95	Tyr62, Leu65, Tyr152, & Trp156	VDW
	Gln96	Arg61	HB
α Framework	Tyr48	His148 & Tyr152	VDW
	Arg66	Asn155	HB
	Arg66	Glu159	SB
CDR2β	Tyr50	Gly68	HB
	Tyr50	Gly68, Gln71, & Met72	VDW
	Asp51	Gln71	VDW
CDR3β	Pro98	Tyr152	VDW
	Ser99	Glu149	HB
	Ser99	His148, Glu149	VDW
	Asn100	Asn146	HB
β Framework	Phe48	Gln64	VDW
	Glu5	Arg67	SB
	Glu56	Gln64 & Arg67	VDW
	Asp5	Arg61	SB
		5-OP-RU	
CDR3α	Tyr95	2′ OH	HB

Atomic contacts determined using the *CONTACT* program of the CCP4i package with cutoff of 4 Å.

Hydrogen bond (HB) interactions are defined as contact distances of 3.5 Å or less.

Van der Waals (VDW) interactions are defined as nonhydrogen bond contact distances of less than 4 Å.

Salt bridge (SB) interactions are defined as contact distances of 4.5 Å or less.

### Role of MAIT TRBV28 **β**-chains and their CDR**β** loops in MR1 recognition

**Figure 5:**
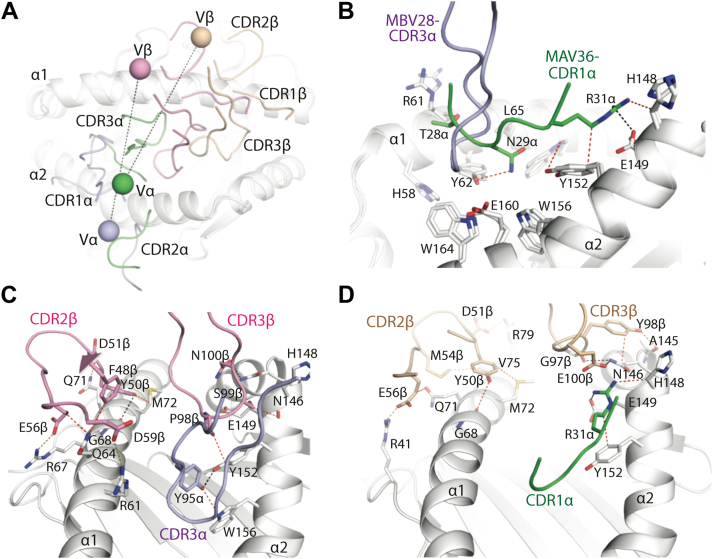
**Interface comparison of TRBV28^+^ TCRs in complex with MR1–5-OP-RU.***A*, superposition of the CDR loops from the TRBV28^+^ TCRs, MAV36 TCR (CDRα, *green*; CDRβ, *wheat*), and MBV28 TCR (colored as in Fig. 4), and superposition of the center of masses of the Vα and Vβ domains above MR1 for MAV36 (Vα, *green sphere*; CDRβ, *wheat sphere*), and MBV28 TCRs (colored as in Fig. 4). *B*, superposition of the CDR1α loop of the MAV36 (*green*) and CDR3α MBV28 (*light blue*) TCRs in complex with MR1–5-OP-RU. Interactions of the CDR1α loop of the MAV36 TCR with MR1 residues are shown. *C*, interaction of the MBV28 TCR β-chain with MR1, and a key interaction with MR1 mediated by the CDR3α loop. *D*, interactions of the MAV36 TCR β-chain with MR1, and a key interaction with MR1 mediated by the CDR1α loop. Hydrogen bonds are represented as *black dashes*; salt bridges as *yellow dashes*; and van der Waals contacts are represented as *dotted red lines*. CDR, complementarity-determining region; MR1, major histocompatibility complex class I–related molecule; 5-OP-RU, 5-(2-oxopropylideneamino)-6-D-ribitylaminouracil; TCR, T-cell receptor.

The MBV28 TCR-β chain interacted exclusively with MR1. Within the TRBV28–TRBJ2-1^+^ TCR-β chain, the CDR3β (14.6% BSA) and CDR2β (13.9% BSA) loops participated roughly to an equal extent at the interface, followed by FWβ (11.1% BSA), whereas the CDR1β loop was essentially not involved in contacts (0.3% BSA) (Fig. 3*D*). The structural determinants of TRBV28 selection were attributable to a combination of germline-encoded residues, namely Tyr50β and Asp51β of CDR2β and Phe48β, Glu56β, and Asp59β of FWβ. Here, the CDR2β motif interacted with MR1 residues, Gly68, Gln71, and Met72, and the FWβ residues, Glu56β and Asp59β, formed salt bridges with MR1 residues, Arg67 and Arg61, respectively, and Phe48β contacted Gln64 of MR1 (Fig. 5*C*). The CDR3β loop of the MBV28 TCR interacted with residues in the MR1 α2-helix spanning 146 to 152, where Pro98β interacted with MR1-Tyr152, Ser99β is H-bonded with Glu149, and formed van der Waals contacts with MR1 residues, His148 and Glu149. Further, the germline-encoded TRBJ2-1^+^ residue, Asn100β, formed a hydrogen bond with MR1-Asn146 (Fig. 5*C* and Table 2). Compared with the MBV28 TCR, the TRBV28^+^ of the MAV36 TCR is tilted closer to the MR1 α1-helix and consequently forms additional CDR2β-mediated salt bridge interactions *via* Glu56β and Asp51β with MR1 residues, Arg41 and Arg79, respectively (Fig. 5*D*). Like the *trbj2-1*-encoded Asn100β residue of the MBV28 TCR, the MAV36 TCR *trbj*-encoded CDR3β residue, Glu100β, formed H-bonds with the MR1-Asn146 residue located at the hinge region of the α2-helix (Fig. 5, *C* and *D*).

These structural data showed that TRAV1-2–TRBV28 MAIT TCR can bind riboflavin derivative–based ligands presented by MR1 through its germline-encoded CDR1α, CDR2α, CDR3α, CDR2β, FWβ, and CDR3β loops. These findings also explain the selection of the semi-invariant MAIT TRAV1-2–TRAJ33 and the TRBV28–TRBJ2 gene segments.

## Discussion

Central to the MAIT cell lineage is expression of a semi-invariant TCR, which, during intrathymic selection, instructs the hallmark characteristics that define these cells (3, 35, 36). While the vast majority of MAIT cells express a TRAV1-2^+^ TCR, rearrangement of a TRAV36–TRAJ34 TCR-α chain with a TRBV28–TRBJ2-5 TCR-β chain generates a distinct semi-invariant TCR with similar MR1–Ag reactivity and phenotypic features that match the defining characteristics of the MAIT cell lineage (17, 29). Here, biochemical and structural analyses were performed to further understand and compare the molecular basis for MR1–Ag recognition by diverse MR1-restricted TCRs, with a focus on the shared TRBV28 TCR-β chain.

TRAV1-2^+^ and TRAV36^+^ MR1-restricted TCRs utilize distinct docking modes atop MR1, thus requiring distinct molecular interactions to facilitate ribityl Ag recognition (17). While TRAV1-2^+^ MAIT TCRs dock in a manner that projects the *traj* gene–encoded Tyr95α of the semi-invariant CDR3α loop into the Ag-binding cleft to make an H-bond with the ribityl tail of the 5-OP-RU (13), 5-OP-RU recognition by TRAV36^+^ TCRs is mediated by Asn29α encoded within CDR1α of the *trav36* gene (17). Similar to TRAV1-2^+^ TCRs however, in which mutation of Tyr95α to alanine or phenylalanine completely ablates Ag recognition, in the present study, mutation of Asn29α in the TRAV36^+^ TCR also resulted in a complete loss of Ag reactivity. Thus, although *via* distinct mechanisms, both classes of MR1-reactive TCRs converge upon a single H-bond to mediate microbial Ag recognition. Only two other human *trav* genes encode an asparagine at position 29, *trav2* and *trav24*; however, other regions of the TRAV36 protein involved in MR1 docking are not conserved across these other *trav* genes (37), providing a basis for the unique use of TRAV36. Mutagenesis data presented here also demonstrate a strong dependence on multiple *traj34*-encoded residues within the CDR3α loop of the TRAV36 TCR for MR1–Ag recognition, thereby explaining the semi-invariant, germline-encoded nature of TRAV36^+^ MAIT TCRs. Moreover, our previous work demonstrated that some TRAV36^+^ TCRs can utilize the *traj37* gene. This is the only other *traj* gene to maintain two of these residues in the same position (37), namely asparagine and threonine, which, when rearranged with *trav36*, were conserved in TCR-α chain positions 93α and 94α, respectively (29). This is akin to the conservation of Tyr95α across *traj12*, *traj20*, and *traj33* within the TRAV1-2^+^ MAIT TCR repertoire (11).

While extensive variation in *trbv* gene usage is permitted within the TRAV1-2^+^ MAIT repertoire, albeit with enrichment of *trbv6-1*, *trbv6-4*, and *trbv20-1*, the TRAV36^+^ TCR repertoire has a high dependence on the *trbv28* gene recombined with *trbj2–5*, with limited CDR3β length (29). Alanine-scanning mutagenesis data performed here suggest only a weak reliance on *trbj2–5*-encoded Gln99β, which forms an H-bond with Tyr92α, perhaps playing a role in stabilizing the CDR3α loop. On the other hand, H-bonds observed between CDR3β and MR1 (17) are neither germline encoded nor conserved across the broader repertoire (29). Irrespective of the molecular basis for this conservation, however, this may explain the low frequency of TRAV36^+^ relative to TRAV1-2^+^ MAIT cells where far greater diversity is permitted within the TCR-β chain repertoire.

Of the MAIT TCR–MR1-Ags ternary complexes published to date, these TCRs all utilize the enriched TRBV from the TRBV6 or TRBV20 family, yet the MAIT TCR repertoire can utilize a range of less commonly used *trbv* genes; how these different β-chains affect MAIT TCR docking on MR1 is unknown. In this study, the crystal structure of a TRAV1-2^+^ MAIT TCR with a TRBV28 TCR-β chain, clone MBV28, in complex with MR1–5-OP-RU was determined. This offers molecular insight into MAIT TCR recognition in the context of an atypical TRBV segment and facilitates a direct comparison with a TRAV36^+^ TRBV28^+^ TCR. Despite shared *trbv* usage, the docking mode of the TRAV1-2^+^ MAIT TCR closely resembles the approximate orthogonal binding mode atop MR1–5-OP-RU utilized by other TRAV1-2^+^ MAIT TCRs, differing markedly from the TRAV36^+^ TCR (8, 13, 14). Indeed, between the two TRBV28^+^ TCRs, the TRBV28 TCR-β chains adopted distinct docking orientations and interacted with a different set of amino acids along the α1 and α2 helices of MR1, thus suggesting that the invariant TCR-α chains of the two TCRs drive the interaction and dictate the overall docking mode. Despite these distinct modes of recognition, binding studies performed here demonstrated similar affinities for MR1–5-OP-RU between the two TCRs, albeit these affinities were moderately lower relative to other TRAV1-2^+^ TCRs utilizing the more frequent *trbv* genes (14) such as *trbv6-1*, measured here. This is potentially, at least in part, a result of the lower contribution to docking by TRBV28 as compared with the more frequently used TRBV6-1, TRBV6-4, and TRBV20-1 TCR-β chains as well as displacement toward the F′ pocket required to permit TRBV28 binding as observed in the crystal structure presented here.

In summary, this research provided 1) molecular insights into MR1 recognition by the invariant MAIT-like TRAV36^+^ population, 2) the basis of MAIT TCR recognition within the framework of an atypical TRBV28 segment, and 3) an understanding of how a single TRBV β-chain can be utilized by two TCRs exhibiting different docking modes. This study collectively emphasizes the central nature of the TCR-α chain from both the TRAV1-2^+^ and TRAV36^+^ repertoires in facilitating Ag recognition. Further questions remain as to whether the differences in TRAV1-2^+^ and TRAV36^+^ TCR docking modes may result in distinct antigenic repertoires, as mediated *via* CDR3β diversity or their divergent positioning of the CDR1α and CDR3α loops. The public nature of both types of TCR, where distinct TCR-α and TCR-β chains provide molecularly distinct, yet convergent, reactivity to MR1–5-OP-RU suggests redundancy in the MR1-restricted T-cell arm of immunity ensuring the ability to recognize microbial Ags presented by MR1. As the number of defined MR1 ligands grows, it will be important to test antigenicity for both TRAV1-2^+^ and TRAV36^+^ MR1-reactive TCRs, as well as to track both subsets in the settings of human health and disease.

## Experimental procedures

### MR1-restricted ligands

Ac-6-FP (catalog no.: 11.418) was synthesized by Schircks Laboratories. Methylglyoxal was purchased from Sigma–Aldrich. 5-A-RU and 5-OP-RU were synthesized as previously described (38).

### Flow cytometry

Single cell suspensions of HEK293T cells or Jurkat 76 cells were stained in fluorescence-activated cell sorting (FACS) buffer consisting of PBS supplemented with 2% fetal bovine serum (Thermo Fisher Scientific). HEK293T cells were stained for 15 min at room temperature with PE-labeled MR1–5-OP-RU tetramers (produced in-house as per (29)), mouse antihuman CD3ε antibodies (Clone UCHT1; BD Pharmingen; catalog no.: 555333; 1:50 dilution), or mouse antihuman Vβ3 (Clone JOV1.3; BD Pharmingen; 566432; 1:50 dilution) antibodies as well as Live/Dead Fixable Near IR viability dye (Thermo Fisher Scientific). Jurkat cells were stained for 30 min at 4 °C simultaneously with PE-labeled MR1–5-OP-RU tetramers, antihuman CD3ε BV421 antibodies (Clone UCHT1; BioLegend; catalog no.: 526426; 1:200 dilution), and Live/Dead Fixable Near IR viability dye in staining solution premixed with titrating doses of soluble TCR protein as described later. After staining, both HEK293T and Jurkat cells were washed once, fixed in 2% paraformaldehyde (PFA) for 10 min at room temperature before a final wash prior to acquisition *via* flow cytometry. Samples were acquired on an LSR Fortessa flow cytometer (BD Biosciences). Data were analyzed using FlowJo Software (Treestar). Cells were gated on viability dye negativity, a homogenous profile of forward scatter (FSC)-A and side scatter-A with subsequent doublet exclusion using FSC-A and FSC-H. HEK293T cells were then gated for a set “window” of PE fluorescence on the Vβ3-stained cells to achieve an equivalent MFI for TCR expression across all transfected cells, as described in Figure 1. This gate was then applied to the MR1–5-OP-RU and anti-CD3ε-stained samples for a given transfection. Jurkat cells were gated on a set “window” of CD3/GFP coexpression across all samples, and the MFI of MR1–5-OP-RU tetramer PE fluorescence was calculated on this gated population.

### Cloning of mutant MAV36 TCR constructs

Double-stranded DNA encoding the full-length MAV36 TCR-α chain or MAV36 TCR-β chain furnishing single point mutations was synthesized between EcoRI and BamHI or XmaI and XhoI, respectively (Thermo Fisher Scientific). DNA was cloned directly into an existing pMIGII construct encoding the wildtype MAV36 TCR (17) using the respective restriction enzymes, thereby replacing the wildtype TCR-α or TCR-β chains with single-alanine point mutants. Novel plasmids were sequence verified *via* Sanger sequencing (Australian Genome Research Facility).

### Transient transfections

HEK293T cells were cultured in a complete medium consisting of RPMI1640 base (Thermo Fisher Scientific), supplemented with 10% fetal bovine serum (Thermo Fisher Scientific), penicillin (100 U/ml), streptomycin (100 μg/ml), GlutaMAX (2 mmol/l), sodium pyruvate (1 mmol/l), nonessential amino acids (0.1 mmol/l), Hepes (15 mmol/l), pH 7.2 to 7.5 (all from Thermo Fisher Scientific), and 2-mercaptoethanol (50 mmol/l; Sigma). Cells were transfected as previously (17). In brief, Fugene6 transfection reagent (Promega) was used to cotransfect cells with pMIGII plasmids encoding the human CD3ε, δ, γ, and ξ subunits linked by p2a-linkers, as well as p2a-linked full-length TCR-α and TCR-β chains encoding the MAV36 TCR or mutants thereof. Cells were incubated for 72 h before being harvested mechanically, passed through a 70 μm strainer, and washed in FACS buffer as above prior to staining for flow cytometry.

### Tetramer inhibition assays

MR1–5-OP-RU tetramers and antibody cocktails were mixed with soluble TCR in twofold dilution starting at a top dose of 1 mg/ml in 50 μl FACS buffer for 15 min at room temperature. Jurkat.M33-64 cells (previously described (17)) were then stained as described previously.

### SKW-3 coculture assays

SKW-3.TCR cells and C1R.MR1 mutants were described previously (11, 17). SKW-3 cells were labeled with Cell Trace Violet and subsequently coincubated with C1R cells and 2 nM 5-OP-RU for approximately 20 h prior to harvest. Cells were then stained with anti-CD69 PE-Cy7 (Clone FN50; BD Pharmingen; catalog no.: 557745) and Live/Dead Fixable Near IR viability dye for 30 min at room temperature, washed three times, fixed with 2% PFA, and analyzed by flow cytometry.

### TCR tetramer staining

C1R cells were incubated in complete medium as aforementioned for 5 h with 10 μM 5-OP-RU or Ac-6-FP (Fig. 2*E*) or 1 μM for 3 h with 5-OP-RU (Fig. 2*G*). Cells were then stained with PE-labeled TCR tetramers, anti-MR1-PE (Clone 26.5; BioLegend; catalog no.: 361106) and Live/Dead Fixable Near IR viability dye for 30 min at room temperature, washed twice and fixed in 4% PFA for 10 min prior to analysis by flow cytometry.

### Recombinant expression and purification of soluble proteins

Soluble MBV28, A-F7, and M33-64 MAIT TCR and human MR1–β-2-microglobulin (β2m) were folded from inclusion bodies *via* oxidative refolding and purified using methods based on those previously described (8, 17). In brief, MR1, β2m, TCR-, and TCR-β chains of the TCR were overproduced separately as insoluble inclusion bodies in *Escherichia coli* BL21(DE3) cells that had been transformed with pET30 plasmid containing the gene of interest. For the TCRs, equimolar quantities of TCR-α : TCR-β chain inclusion bodies were placed in refold buffer consisting of 100 mM Tris (pH 8.0), 2 mM EDTA, 0.4 M l-arginine–HCl, 5 M urea, 0.5 mM oxidized glutathione, 5 mM reduced glutathione to a final pH 8.5, in the presence of PMSF and pepstatin A. In addition, MR1–β2m bound to 5-OP-RU was refolded by sequential dilution of MR1 and β2m inclusion bodies into refold buffer as aforementioned but at pH 8.0, with the addition of methylglyoxal and 5-A-RU, as described previously (8). Refolds were incubated overnight at 4 °C with stirring, before 24 h of dialysis in 10 mM Tris prior to purification. Protein of interest was purified using weak anion exchange (DEAE Sepharose; GE Healthcare), size-exclusion (Superdex 75; GE Healthcare), and strong anion exchange (MonoQ; GE Healthcare) chromatography, as previously described (17). TCR tetramers were produced as aforementioned using a TCR-α chain construct with a C-terminal AVI tag for enzymatic biotinylation using birA enzyme. Biotinylated TCRs were further purified using size-exclusion chromatography (Superdex 200; GE Healthcare). All proteins were eluted in 10 mM Tris (pH 8), 150 mM NaCl, and stored at −80 °C. Protein purity was determined by SDS-PAGE, and concentrations were calculated from absorbance values at 280 nm using a NanoDrop spectrophotometer.

### Surface plasmon resonance

All SPR experiments were conducted in duplicate and n = 2 independent experiments were performed at 25 °C on a BIAcore 3000 instrument using HBS buffer: 10 mM Hepes (pH 7.5), 150 mM NaCl, and 0.005% surfactant P20 (GE Healthcare) (16). Biotinylated C-terminal cysteine-tagged-MR1–Ag generated as previously described were immobilized on a streptavidin sensor chip with ∼2000 response units (14). The analyte TCR was injected over the captured MR1–Ag at a flow rate of 5 μl/min. The final response was calculated by subtracting the response of the blank flow cell alone from the TCR–MR1–Ag complex. The SPR sensorgrams, equilibrium curves, and steady state affinity *K*_*D*_ values (μM) were prepared using GraphPad Prism.

### Crystallization, structure determination, and refinement

Crystals of the soluble MBV28 TCR–MR1–5-OP-RU complex were obtained using the hanging drop vapor-diffusion method at 20 °C. The purified MR1–β2m–5-OP-RU and MBV28 TCR were mixed in a 1:1 M ratio to a final concentration of 4 mg/ml, then equal volumes of protein complex to precipitant solution consisting of 0.2 M sodium acetate, 0.1 M Bis–Tris propane (pH 6.1 to 6.5), and ∼12 to 18% PEG 3350 was added. Crystals were cryoprotected before diffraction experiments by soaking in the mother liquor supplemented with 10 to 15% glycerol before flash freezing in liquid nitrogen. Diffraction images were collected on the MX2 beamline at the Australian Synchrotron using the EIGER X 16M pixel detectors at 100 K (39). The data were processed using XDS package (MPI for Medical Research) (40) and scaled using *Aimless* in the CCP4 suite (41).

The crystal structure of MBV28–MR1–5-OP-RU was solved by molecular replacement using PHASER in the Phenix suite (42), using A-F7–MR1–5-OP-RU (Protein Data Bank [PDB] entry: 6PUC) without CDR loops and in the absence of 5-OP-RU as the initial search model. This structure was refined by iterative rounds of manual adjustment in Coot (Crystallographic Object-Oriented Toolkit; developed by MRC Laboratory of Molecular Biology) and restrained refinement in Phenix. Only one ternary complex was present in the asymmetric unit. The restraints for 5-OP-RU were produced using the Grade Web Server, with building performed in Coot. Final refinement statistics and PDB entry are summarized in Table 1. Validation of models was achieved using MolProbity, and all graphical representations were generated using the PyMOL Molecular Graphics System, version 2.5. The BSA was calculated using Areaimol in the CCP4 suite (41).

## Data and code availability

Data and reagents are available upon reasonable request. The coordinates and structure factor for MBV28–MR1–5-OP-RU have been deposited at the PDB (www.rcsb.org) with accession number “9BYS.”

## Supporting information

This article contains supporting information.

## Conflict of interest

L. L., D. P. F., J. M., and J. R. are inventors on patent applications (PCT/AU2013/000742, WO2014005194; PCT/AU2015/050148, WO2015149130) describing MR1 ligands and MR1-tetramer reagents. All other authors declare that they have no conflicts of interest with the contents of this article.
