# Supplementary material for: A molecular basis underpinning TRBV28+ T-cell receptor recognition of MR1–antigen
Source: J Biol Chem. 2025 Jun 24;301(8):110416. doi: 10.1016/j.jbc.2025.110416 (PMC12309606; doi:10.1016/j.jbc.2025.110416)
Supplement: Supplementary Material [file mmc1.pdf]

A.

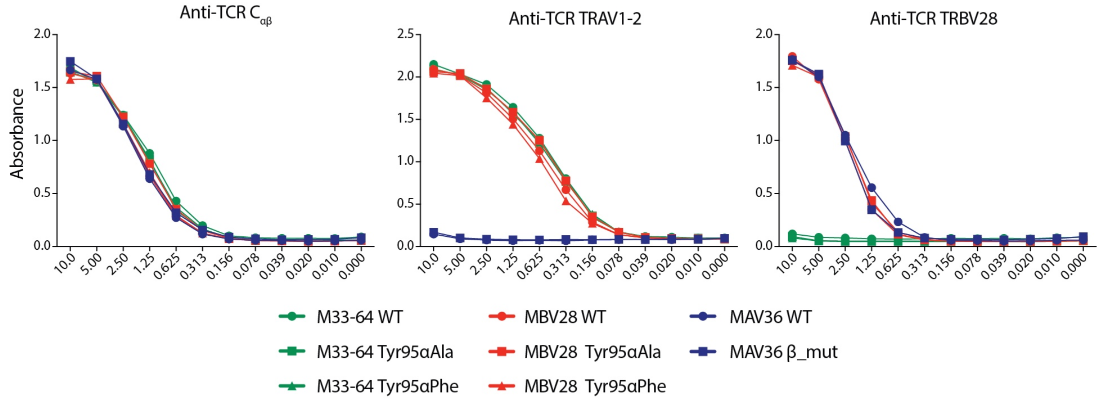

B.

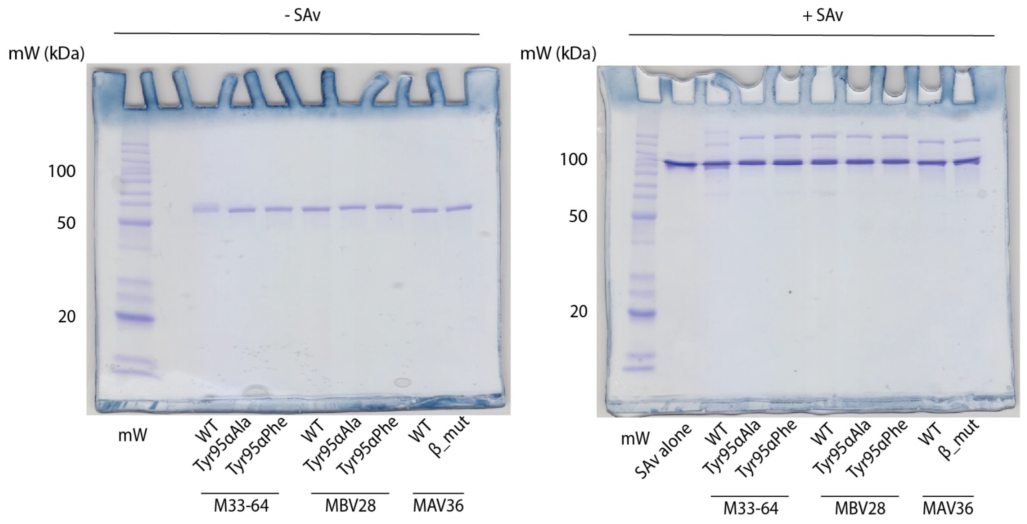

Figure S1. A. Line graphs of ELISAs showing anti-TCR Cαβ, anti-TRAV1-2 and anti-TCR TRBV28 antibody binding to biotinylated TCRs. B. SDS-PAGE gels confirming biotinylation of TCR proteins as demonstrated by higher apparent molecular weight in the presence of streptavidin (right) compared to no streptavidin (left).

Figure S1
